# Do Extreme Values of Daily-Life Gait Characteristics Provide More Information About Fall Risk Than Median Values?

**DOI:** 10.2196/resprot.3931

**Published:** 2015-01-05

**Authors:** Sietse M Rispens, Kimberley S van Schooten, Mirjam Pijnappels, Andreas Daffertshofer, Peter J Beek, Jaap H van Dieën

**Affiliations:** ^1^MOVE Research Institute AmsterdamFaculty of Human Movement SciencesVU University AmsterdamAmsterdamNetherlands

**Keywords:** accidental falls, risk assessment, accelerometry, activities of daily living, gait, walking, logistic models, aged

## Abstract

**Background:**

Gait characteristics estimated from daily-life trunk accelerations reflect gait quality and are associated with fall incidence in older adults. While associations are based on median values of these gait characteristics, their extreme values may reflect either high-risk situations or steady-state gait and may thus be more informative in relation to fall risk.

**Objective:**

The objective of this study was to improve fall-risk prediction models by examining whether the use of extreme values strengthens the associations with falls.

**Methods:**

Trunk acceleration data (Dynaport MoveMonitor) were collected from 202 older adults over a full week. From all walking episodes, we estimated the median and, as reliable estimates of the extremes, the 10th and 90th percentiles of gait characteristics, all over 10-second epochs. In addition, the amount of daily activities was derived from the acceleration data, and participants completed fall-risk questionnaires. Participants were classified as fallers based on one or more falls during 6 months of follow-up. Univariate analyses were performed to investigate whether associations with falls were stronger for the extremes than for the medians. Subsequently, three fall-risk models were compared: (1) using questionnaire data only, (2) adding the amount of activities and medians of gait characteristics, and (3) using extreme values instead of medians in the case of stronger univariate associations of the extremes.

**Results:**

Stronger associations were found for the extreme characteristics reflecting high regularity, low frequency variability, and low local instability in anterior-posterior direction, for high symmetry in all directions and for low entropy in anterior-posterior and vertical directions. The questionnaire-only model improved significantly by adding activities and gait characteristics’ medians. Replacing medians by extremes with stronger associations did improve the fall prediction model, but not significantly.

**Conclusions:**

Associations were stronger for extreme values, indicating “high gait quality” situations (ie, 10th and 90th percentiles in case of positive and negative associations, respectively) and not for “low gait quality” situations. This suggests that gait characteristics during optimal performance gait provide more information about the risk of falling than high-risk situations. However, their added value over medians in prediction is limited.

## Introduction

Identifying persons with a high risk of falling can facilitate effective prevention of falls. Several ways of assessing fall risk have been investigated, including questionnaires, physical tests, gait analysis, and physical activity measurements [1-4]. Yet the predictive value of these models is still limited.

A promising way to assess fall risk is by means of body-worn sensors in daily life. Trunk accelerations during walking can provide information about personal risk factors for falls related to physical capacity and health status. This information is typically assessed by gait analysis in controlled settings, which has shown that high variability, low stability, and low symmetry of gait are associated with falling [5,6]. The use of body-worn sensors in daily life can add information about physical activity [7], as well as situational fall-risk factors related to one’s behavior and environment [8]. This new approach has demonstrated the potential to make an important contribution to fall-risk assessment, as shown by daily-life gait characteristics’ associations with falls and the added value of gait characteristics in fall prediction over commonly used methods [9-11].

Even though the previously developed fall-risk model based on daily-life gait characteristics showed a very promising performance (ie, an area under the receiver operator curve [AUC] of 0.82) [10], there may still be room for improvement. One aspect to consider is the selection of specific gait episodes in daily life that contain the most relevant information for fall-risk prediction. Previous studies used the mean or median of a gait characteristic over the analyzed epochs of gait, based on the assumption that this would be the most representative estimate for that characteristic [9-11]. However, as situations in daily life vary, gait characteristics obtained in particular situations may better reflect a person’s fall risk than the median of those obtained in all analyzed epochs of gait. On the one hand, episodes with “low gait quality” may contain information about taking risks or responding to risks in such situations. High-risk situations may be expected to show high variability and low stability and symmetry, as these gait characteristics are associated with falling [5,6,9-15]. On the other hand, situations where people show “high gait quality” might be informative about the best possible performance they can achieve, which may be closely related to personal risk factors or the performance in a lab or on a treadmill. We expected these two extreme situational effects, high-risk situations and optimal gait performance, to be reflected in the extreme values of gait characteristics calculated over 1 week. Therefore, we investigated whether extreme values of gait characteristics obtained in daily life had stronger associations with falls and predicted these falls better than their median values.

## Methods

### Participants

The 202 individuals who participated in this study were part of the larger Fall Risk in Old Age (FARAO) cohort study conducted at VU University Amsterdam. They were mainly community dwelling older adults, and inclusion criteria were having a mini mental state examination (MMSE) of at least 19 (out of 30 points), age between 65 and 99, and the ability to walk 20 meters continuously, with the aid of an assistive device if needed. All participants provided written informed consent and the medical ethical committee of the VU University Medical Hospital approved the protocol (number 2010/290).

### Protocol

At the start of the study, participants were interviewed and trunk accelerations were recorded over a full week, after which their fall incidence was monitored for 6 months. During the interview, demographic information was collected (Table 1), as well as fall history and the geriatric depression score (GDS) [16], since these were previously shown to be associated with future falls [10]. Following the interview, participants wore a tri-axial accelerometer (MoveMonitor, McRoberts, The Hague, Netherlands; sampling range -6g to 6g; sampling rate 100 Hz) for 1 week continuously, except during water-related activities that could damage the device. Participants were instructed to wear the accelerometer with an elastic band around their waist at the mid-back, at the level of their lumbar spine. After the interview and measurements, participants’ falls were monitored for 6 months by monthly telephone contact in addition to a daily diary. If participants had fallen at least once during the 6-month follow-up, they were classified as fallers; if not, they were classified as non-fallers.

**Table 1:** Participant demographics.

	Fallers (n=70)	Non-fallers (n=132)	*P* (*t* test)
Age (years), mean (SD)	75.6 (6.1)	75.1 (6.6)	.59
Gender (male), %	47	50	.73
Community dwelling, %	94	94	.93
Weight (kg), mean (SD)	75 (14)	74 (14)	.45
Height (m), mean (SD)	1.71 (0.09)	1.70 (0.09)	.46
BMI (kg/m^2^)	25.6 (3.7)	25.3 (3.6)	.57
Use of walking aid, %	26	22	.66
MMSE (max 30), mean (SD)	27.7 (2.3)	27.8 (2.1)	.78

### Physical Activities and Gait Characteristics

The accelerations recorded during the measurement week were used to estimate the average amounts of physical activities as well as a comprehensive set of gait characteristics. Table 2 (gait characteristics) and Table 3 (physical activities) cover the complete set of derived parameters. The parameters in question were estimated as described in previous papers (see [7,10] for physical activity and [9,17] for gait characteristics). We added the estimation of sample entropy [18,19] in view of its potential to discriminate fallers from non-fallers [10,20]. We used 5 consecutive data points and 0.3 as the radius of tolerance, based on the determination of auto-regressive process orders and relative errors of sample entropy for our data as proposed by Lake et al [21]. In order to focus solely on regular walking, we discarded locomotion episodes suspected to reflect running. These episodes, which caused severe outliers for some participants, were identified by a low stride time (<0.8 s) and/or a high vertical (VT) acceleration root-mean-square (RMS) (>5 ms^-2^).

As in previous studies, median values of gait characteristics were estimated from all 10-second walking epochs recorded during the measurement week. In addition, the extremes were estimated as the parameter values of the 10th and 90th percentiles of the gait characteristics derived. We used the 10th and 90th percentile values as best estimates of the extremes themselves, since the reliability of the extremes appeared to be insufficient; when using the data and procedures as described in previous work [9], but now for extremes instead of medians, more than 90% of the between-weeks intraclass correlations (ICC) were below 0.7. For the 10th and 90th percentile values, more than 90% had an ICC above 0.7, similar to the medians.

### Statistical Analysis

#### Univariate Regression

We assessed the added value of extreme values of gait characteristics over median values by comparing their association with fall incidence through univariate logistic regression. A percentile value (10th, 50th, or 90th) was considered to have a stronger association over the other two percentile values when its regression *P* value was lowest and below .05.

#### Generating Fall Prediction Models

Three fall-risk models were generated. Model 1 was based on the participant’s fall history (yes/no) and the GDS score. Other data obtained through the interview were not used for the fall prediction models because these were not associated with falling in a previous study [10]. In Model 2, all amounts of physical activities were added, as well as the median values of all gait characteristics. In Model 3, we replaced the median value of a given gait characteristic by its extreme value, provided that the latter had a stronger association with falls according to the univariate analysis. The outcome variable for all three models was whether or not participants had fallen at least once during the 6 months of follow-up. The fall prediction models were generated by means of stepwise forward logistic regression. In every step, the parameter with the lowest *P* value below .05 when added to the parameters in previous steps was selected, provided that the parameter did not have an absolute Spearman correlation higher than .7 with any of the previously selected parameters. The models were tested for inadequate calibration between predicted probabilities and observed fall incidences using the Hosmer-Lemeshow test.

#### Comparing Fall Prediction Models

When evaluating the added value of new parameters for a prediction model, it is not trivial to determine the significance of added parameters while considering the number of parameters that were available for selection. Commonly used tests typically compare pre-determined prediction parameters (eg, [22,23]) and do not account for the freedom of parameter selection or the setting of regression coefficients. In this study, to estimate a *P* value for the improvement of the models when adding or replacing parameters, we required a test that could handle models with different numbers of parameters and could select parameters from subsets of different numbers of potential parameters. Since we found no analytical test that satisfied these requirements, we used a Monte Carlo permutation test. All parameters as obtained from the week of acceleration data collected from one participant were permuted with the data of another randomly selected participant. Since all gait characteristics and amounts of activities were taken from the same week of accelerations of another participant, correlations between these parameters remained the same. Questionnaire data and fall incidence were not permuted between participants. We generated models for 1000 differently permuted datasets and estimated the *P* value for the improvement between models as the ratio of permutations having a larger increase of the area under the receiver operator curve (AUC) between the models than the increase of the AUC between the models obtained with the original dataset. The first model, being based exclusively on questionnaire data, was not affected by the permutations.

## Results

Univariate regression showed significant associations (*P*<.05) with falling for 14 out of 30 gait characteristic medians (Table 2). High stride regularity anterior-posterior (AP), high harmonic ratio VT and AP, low local dynamic stability AP, and low sample entropy VT had a stronger association than the medians of these characteristics. When using extremes, the associations of low frequency variability AP, high harmonic ratio mediolateral (ML), and low sample entropy AP with falls were significant, whereas they were not significant when using the median values. All stronger associations for extremes were found for the extremes related to optimal performance gait, that is, the 10th percentile (lower extreme) in case of positive associations with falls, and the 90th percentile (higher extreme) in case of negative associations. Regression results of questionnaire data and amounts of activities showed significant associations for fall history, GDS depression scale, and lying duration (Table 3).

**Table 2:** Univariate logistic regression of gait characteristics’ 10th, 50th, and 90th percentile values, with future falls (B values [*P* values] are based on *z*-transformed data).

Characteristics	10th perc. B (*P*)	Median B (*P*)	90th perc. B (*P*)
Gait speed	-0.36 (.03)	-0.38 (.02)^a^	-0.32 (.04)
Speed variability	0.26 (.10)	0.19 (.21)	-0.08 (.60)
Stride frequency	-0.21 (.15)	-0.31 (.04)^a^	-0.15 (.41)
Frequency variability VT	0.25 (.09)	0.26 (.08)	-0.12 (.41)
Frequency variability ML	0.25 (.10)	0.21 (.15)	0.01 (.96)
Frequency variability AP	0.33 (.03)^a^	0.27 (.07)	0.07 (.61)
Stride regularity VT	-0.23 (.14)	-0.34 (.03)^a^	-0.26 (.08)
Stride regularity ML	0.07 (.65)	-0.08 (.58)	-0.06 (.70)
Stride regularity AP	-0.24 (.12)	-0.32 (.04)^a^	-0.37 (.02)^a^
RMS VT	-0.29 (.07)	-0.50 (.004)^a^	-0.41 (.009)
RMS ML	0.09 (.53)	-0.08 (.60)	-0.06 (.67)
RMS AP	-0.20 (.19)	-0.37 (.02)^a^	-0.25 (.11)
Low frequency percentage VT <0.7 Hz	0.20 (.18)	0.38 (.01)^a^	0.23 (.11)
Low frequency percentage ML <10 Hz	0.24 (.12)	0.33 (.04)^a^	0.15 (.32)
Low frequency percentage AP <0.7 Hz	0.28 (.06)	0.37 (.01)^a^	0.27 (.07)
Index of harmonicity VT	-0.16 (.30)	-0.23 (.12)	-0.20 (.17)
Index of harmonicity ML	0.27 (.07)	0.24 (.11)	0.13 (.37)
Index of harmonicity AP	0.11 (.45)	0.18 (.23)	0.28 (.07)
Harmonic ratio VT	-0.03 (.83)	-0.43 (.009)^a^	-0.51 (.003)^a^
Harmonic ratio ML	0.11 (.46)	-0.25 (.11)	-0.32 (.048)^a^
Harmonic ratio AP	-0.05 (.76)	-0.41 (.01)^a^	-0.53 (.002)^a^
Local dynamic stability VT	0.29 (.053)	0.33 (.03)^a^	0.22 (.16)
Local dynamic stability ML	0.10 (.51)	0.08 (.58)	0.03 (.86)
Local dynamic stability AP	0.38 (.02)^a^	0.34 (.03)^a^	0.27 (.08)
Sample entropy VT	0.60 (<.001)^a^	0.41 (.02)^a^	0.14 (.35)
Sample entropy ML	-0.09 (.54)	-0.12 (.43)	-0.16 (.31)
Sample entropy AP	0.39 (.01)^a^	0.22 (.15)	0.02 (.87)
Dominant frequency’s amplitude VT	-0.22 (.16)	-0.29 (.06)	-0.19 (.21)
Dominant frequency’s amplitude ML	0.16 (.28)	0.14 (.34)	0.15 (.31)
Dominant frequency’s amplitude AP	-0.13 (.37)	-0.06 (.71)	0.07 (.63)

^a^Significant associations of medians and stronger associations of extremes.

**Table 3:** Univariate logistic regression of questionnaire parameters and amounts of physical activities with future falls (B values [*P* values] are based on *z*-transformed data).

	B (*P*)
Fall history	0.55 (<.001)^a^
GDS depression scale	0.45 (.003)^a^
Lying duration	-0.40 (.02)^a^
Sitting duration	0.27 (.09)
Standing duration	0.24 (.11)
Locomotion duration	-0.01 (.96)
Shuffling duration	0.23 (.12)
Number of transitions^b^	0.15 (.30)
Number of steps	-0.05 (.76)

^a^Significant associations.

^b^Direct transitions from sedentary (lying and sitting) to non-sedentary (standing, locomotion, and shuffling) activities.

Fall history was selected as a parameter in all models. GDS depression score was selected in the questionnaires-only model but not in the models including acceleration data. In both models that included acceleration data, lying duration as well as low frequency percentage below 0.7 Hz in the VT and sample entropy in both the VT and ML direction were selected (Table 4). For Model 3, only one extreme value (10th percentile of Sample Entropy VT) was selected as a parameter. The Hosmer-Lemeshow test revealed no indications of inadequate calibration (*P*=.58, .41, and .76 for Models 1-3, respectively).

**Table 4:** Fall prediction models with parameter coefficients and *P* values.

Models	B	*P* value	Parameters
**Model 1: questionnaires only**			
	-0.70	<.001	Constant
	0.46	.004	Fall history
	0.33	.04	GDS depression score
**Model 2: add activities and gait characteristics’ medians**			
	-0.66	<.001	Constant
	0.67	<.001	Fall history
	0.41	.04	Low frequency percentage VT <0.7 Hz
	-0.43	.02	Lying duration
	0.86	.002	Sample entropy VT
	-0.53	.03	Sample entropy ML
**Model 3: replace gait characteristics’ median if extreme has stronger association**			
	-0.68	<.001	Constant
	0.69	<.001	Fall history
	1.02	<.001	Sample entropy VT (Low extreme)
	0.61	.002	Low frequency percentage VT <0.7 Hz
	-0.60	.004	Lying duration
	-0.47	.03	Sample entropy ML

**Figure 1:**
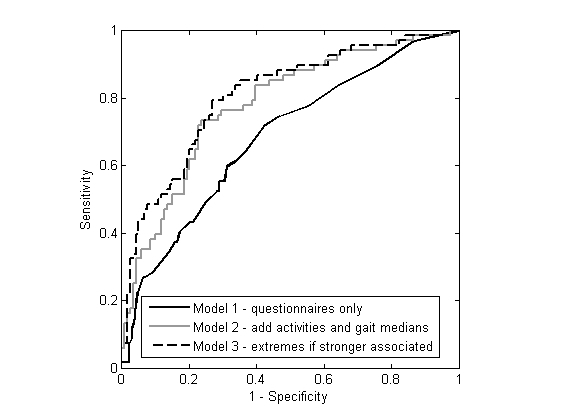
ROC curves for the 3 prediction models.

Receiver operating characteristic (ROC) curves of the 3 prediction models are shown in Figure 1. The AUC for the prediction Models 1-3 were 0.684, 0.781, and 0.808, respectively. Models 2 and 3, which both involved daily-life acceleration parameters, performed significantly better than Model 1 (*P* values for the improvements were .01 and .003, respectively). Model 3 with extremes did not improve significantly with respect to Model 2 based exclusively on median values (*P*=.19).

## Discussion

### Principal Findings

This study investigated whether extreme values of gait characteristics as observed during a single week in daily life are more strongly associated with fall risk than their median values. In particular, we determined the added value for fall-risk assessment of such extreme values of gait characteristics above the median values.

The characteristics with an extreme that had a univariate association stronger than the median, that is, high regularity and harmonic ratio, and low frequency variability, local dynamic stability and entropy, seemed to share a strong dependency on situational effects, as indicated for example by their systematic difference between treadmill and daily-life walking [8]. For all of these characteristics, the strongest associations with fall risk were found for the extremes related to a lower fall risk, which indicates that they were the “high gait quality” extremes. This suggests that for these characteristics the optimal performance in daily-life situations provided more information about fall risk than performance in more demanding situations. These optimal performance gait episodes may be recorded in situations that are more comparable between subjects and may consequently provide a better assessment of an individual’s capacities than the median. However, the most representative values in daily life, as quantified by the median, still had stronger associations for most of the characteristics. The fact that none of the “low gait quality” extremes were found to have a stronger association than the medians suggests that the presence of irregular gait episodes in daily life is not an indication of situations with higher fall risk, but rather an indication of situations that require or permit gait adaptations. Low gait quality extremes may reflect the exposure to environmental constraints, such as imposed by a winding forest track compared to a paved footpath. Moreover, exposed individuals may be those who can cope with such environmental constraints, whereas these are avoided by individuals with acknowledged lower gait quality.

The expected added value of the gait characteristics’ extreme values in daily life above the medians in a fall-risk prediction model was not demonstrated. Although the model including the extremes (Model 3) had a higher AUC than the model with the medians (Model 2), the Monte Carlo permutation test indicated that this difference may have resulted from a combination of chance and an expected improvement due to the replacement of medians with more strongly associated extremes; the *P* value for the improvement found was .19. The improvement of univariate associations found may have been too small to yield a significant effect on the predictive value of the models. Perhaps the high-quality extremes are merely a more accurate estimation of the individual’s capacities and do not provide information about a new concept such as risk-taking behavior. However, the Monte Carlo permutation tests did confirm the previously reported finding that information from trunk accelerations recorded during a week in daily life significantly improves fall prediction models based on questionnaire data alone [10].

### Limitations

The reported AUC and the Monte Carlo permutation test have their limitations. The AUC we reported may have been biased since the AUC was estimated from the same dataset used for the model generation. The presented models should be validated with new or other data, in order to obtain an unbiased AUC. The Monte Carlo permutation test is a method for testing the significance of model improvement by increasing the set of optional parameters. However, in the comparison of Models 2 and 3, we did not add parameters, but rather replaced parameters. It was therefore necessary to compare the change in AUC between permuted and non-permuted data, rather than the AUC itself. This comparison assumed that probability of the improvement of 0.027 (ie, the difference in AUC between Models 2 and 3) is similar for different starting values of the AUC. However, one may assume that such an improvement is less probable when starting with a higher AUC. We can therefore consider the estimated *P* value of .19 as a conservative estimate, since the starting AUC (Model 2) of the permuted data was typically lower than that of the non-permuted data.

### Comparison With Previous Studies

The parameters that were incorporated in the model comparison have been previously linked to falling [1,9,10,20], except for lying duration. Lying duration was negatively associated with fall risk, which might be explained by an elevated risk of falling when one is not well rested, assuming that less lying implies being less well-rested. It may also reflect high fall risk when leaving the bed during the night. Sample entropy was previously found to be associated with falling for the AP direction, but not for the ML direction [20]. Also in this study sample entropy in ML direction was not univariately associated with falling. This hampers the interpretation of its contribution to the model and of its meaning for fall risk in general.

When comparing the results in this study with those reported previously [10], one would expect to find quite similar results since most of the participants (169 out of our 202) participated in both studies. This was indeed the case for the univariate associations. With one exception (local dynamic stability or logarithmic divergence rate in mediolateral direction), all differences in significant associations were still nearly significant with *P*<.10. However, Model 2, which included physical activities and gait characteristics’ medians, contained different parameters than the model derived in the previous study [10]. Apparently, the selection of model parameters was sensitive to slight changes in population. The models were in agreement in that both included fall history and a combination of physical activity parameters and gait characteristics.

### Conclusions

Several “good gait quality” extremes of gait characteristics were estimated to have a stronger association with future falls than their medians. In particular, epochs with low frequency variability and high regularity, symmetry, and stability may be of particular interest for fall-risk prediction. However, using gait characteristics’ extremes in addition to medians did not significantly improve fall prediction models.

## Abbreviations

AP: anterior-posterior
AUC: area under the receiver-operator curve
GDS: Geriatric Depression Score
ICC: intraclass correlation
ML: mediolateral
MMSE: Mini Mental State Examination
ROC: receiver operating characteristic
RMS: root mean square
VT: vertical
